# Understanding the Molecular Basis of iPSC Reprogrammed Cells to Fulfil Their Expectations in Future Clinical Applications

**DOI:** 10.3390/cells11172714

**Published:** 2022-08-31

**Authors:** Verónica González-Fernández, Ana Sevilla

**Affiliations:** 1 Department of Cell Biology, Physiology and Immunology, Faculty of Biology, University of Barcelona, 08028 Barcelona, Spain; 2 Institute of Biomedicine, University of Barcelona (IBUB), 08028 Barcelona, Spain

IPSC-based disease modelling and pluripotency studies have sparked widespread enthusiasm for more than 16 years of research. All this effort and knowledge have presented an unprecedented opportunity in high-throughput drug discovery platforms and safety pharmacology in association with three-dimensional multicellular organoids, personalized organs-on–chips, gene/base editing, 3D chromatin organization, artificial intelligence, and high-throughput “omics” methodologies.

In this Special Issue of *Cells*, four up-to-date reviews on stem cells unravelling the molecular mechanisms of pluripotency to promote somatic cell reprogramming and its differentiation potential for clinical applications are published [1,2,3,4]. Reprogramming patient cell lines have become the gold standard, as these cells continue to find new applications in disease modeling, mechanistic studies, drug development, biobanking, and therapeutic strategies across a diverse range of pathologies [5,6]. However, for iPSCs to be used clinically, the optimization of the starting donor cell type and the reprogramming method must meet certain criteria.

In this Special Issue, Guisseppe Scesa recovers all the information regarding the different reprogramming methods and highlights the presence of certain epigenetic memory of those iPSCs in relation to their tissue of origin which conditions their differentiation efficiency especially at early passages [3,7]. However, broader transcriptional studies regarding the donor cell type, showed that the tissue of origin accounted for less than 4% for the transcriptional variation. In contrast, inter-individual genetic variation was responsible for 38% of the total, with <1% attributable to differences between iPSCs and ESCs [8]. Indeed, differences in gene transcription were maintained throughout differentiation and clustered with the donor rather than with the tissue of origin, causing a variable outcome upon differentiation [9]. This constitutes an important factor to keep in mind since such differences are maintained after transplantation, impacting engraftment and differentiation potential in vivo [10]. Another point of consideration is that the donor cells should be mutation free, accessible, and amenable to reprogramming. Overall, the reprogramming protocol must produce transgene-free iPSCs in a highly efficient manner. There are now a wide range of methods available to generate iPSCs as Guisseppe Scesa extensively described in this Special Issue [3]. The episomal-based vector system, as well as the mRNA system seem to be the most promising reprogramming methods for the clinic. Additionally, to succeed in the reprogramming process, it is desirable to consider the reprogramming protocol, the culture media, the cell type, as well as the age and passage number of the somatic cells. Regarding the culture media conditions, the work performed by Molina-Ruiz et al. commented here, has shown that the standardization of culture conditions and the implementation of a Quality Management System (QMS), such as ISO9001-2015 with routine genomic screening, can significantly decrease the prevalence of genomic alterations affecting hPSCs used for either research applications and clinical transplantation [11].

Another thing to consider is that many of the diseases that will be treated with iPSCs are diseases associated with advanced age like Parkinson, macular degeneration, etc. Ideally, cell replacement therapies will be done with autologous cells although as a counterpart aged cells retain their DNA mutations during reprogramming [12]. For this reason, Mohamed at al. has proposed the use of umbilical cord mesenchymal stem cells (CT_MSC) as another possible source of young cells for the derivation of histo-compatible iPSCs, however those cells will not be autologous [13].

As an alternative, the use of patient mesenchymal stem cells (MSCs) has also been explored as autologous therapy. These cells although they present a limited differentiation potential, able to differentiate into chondrocytes, osteoblasts, adipocytes, tenocytes, or myocytes [14], they have shown utility in specific treatments. Proof of that, is the work presented in this Special Issue by Jacob Mark and collaborators where they explored the generation of induced pluripotent mesenchymal stem cells (iP-MSCs) for autologous transplantation after irradiation treatment because of certain carcinomas [15]. This mesenchymal stem cells are relatively easy to reprogram and considering the epigenetic memory they tend to differentiate back into the mesenchymal estate [16]. Results showed that, although the iP-MSC did not achieved the complete immunological profile of the MSCs regarding IL-6 and IL-8 cytokine release; they reacquired major functional properties, such as suppression of CD4^+^ T cell proliferation.

Although there is still more work to do to fulfill the generalized use of iPSCs in cell replacement therapies, other field where iPSC is becoming quite useful is their use for accelerating drug discovery and personalized precision medicines after many cancer treatments. It is well-known that many cancer drugs, are often poorly tolerated by the body, generating cardiac, liver or kidney toxicity. For this reason, the strategy of biobanking iPSC-derived cells for testing oncologic treatments is raising interest as these cells are human-based, patient-derived, and constitute a genetically variable platform that can be applied to the study of chemotherapy-induced toxicity previously inaccessible through animal models [17,18]. Dr Lee’s group presents here a great revision about the cardiotoxicity of antineoplastic therapies and the use of iPCs derived cardiomyocytes for personalized therapies [1]. Therefore, patient-derived iPSCs show great potential in the field of personalized medicine although future efforts should focus on the improvement of the iPSCs derived cells to resemble more derived accurately mature cells.

To solve this lack of maturity in the iPSC-derived cells, many studies are deepening about the impact of reprogramming methods and how the cell resets the non-GpG methylation patterns during the reprogramming process [19]. In particular, Andreas Hörnblad thoroughly reviews the chromatin accessibility, enhancer function and 3D chromatin organization in the process of reprogramming to pluripotency. All these kind of studies have allowed us to start identifying non-coding regulatory elements in the genome (e.g., enhancers) that control cell-type specific gene expression. The activation of poised ESC-specific enhancers early in reprogramming seems to allow a quick switch to the active enhancer state at later stages of reprogramming when it is crucial to efficiently coordinate the activation and expression of ESC-specific genes. Although what determines the functional interactions between regulatory factors and their target sequences in the genome is still not completely understood.

For this reason, basic and translation stem cell research need to go together as the deciphering of the regulatory mechanisms in stem cells through multi-omics assays holds the key to understand its differentiation potential [20,21]. Nowadays, many studies are being focused on methylation as histone methylation governs gene expression programs. Indeed, methylases play an important role in development controlling the balance between self-renewal and differentiation, which could give us some hits about the reprogramming process. As an example, Dr. Aguiló brings us all the functional details of the Lysine-specific demethylase 1 (LSD). The capability of LSD1 to interact with many other proteins explains the plethora of cellular processes in which this protein participates [2]. In particular, in stem cells, although LSD1 is not essential for ESC self-renewal it is required for differentiation [22]. LSD1 is poised at the enhancers of pluripotent factors where p300/HAT inhibits its activity and, therefore, transcription can occur. During differentiation H3K4me1 LSD1-mediated demethylation, switches off the expression of pluripotent factors. Understanding these cellular processes will enhance our differentiation and maturation efficiency. In summary, coupling iPSC progress with other technologies, such as Crispr-Cas9, 3D organoids, and microRNA switches, will farther advanced the already rapid pace of iPSC-based disease modeling and therapeutic development. Accumulating data of cellular phenotypes of iPSC models from a cross-sectional variety of diseases will significantly contribute to new stratifications and deep understanding the different diseases, which could also lead to new cross-sectional treatment approaches for personalized medicine.
